# Comparison of SaCoVLM™ video laryngeal mask-guided intubation and i-gel combined with flexible bronchoscopy-guided intubation in airway management during general anesthesia: a non-inferiority study

**DOI:** 10.1186/s12871-022-01843-x

**Published:** 2022-09-22

**Authors:** Chun-ling Yan, Yi-qi-yuan Zhang, Ying Chen, Zong-yang Qv, Ming-zhang Zuo

**Affiliations:** Department of Anesthesia, Beijing Hospital, National Center of Gerontology, Institute of Geriatric Medicine, Chinese Academy of Medical Sciences, Beijing, People’s Republic of China

**Keywords:** SaCoVLM™ video laryngeal mask, SaCoVLM™ guided intubation, i-gel, i-gel combined with FB, Supraglottic airway device guided intubation

## Abstract

**Background:**

When a difficult airway is unanticipatedly encountered and the initial laryngoscopic intubation fails, a supraglottic airway device (SAD) may be placed to aid ventilation and oxygenation, and act as a conduit for intubation. SaCoVLM™, as new SAD, can offer a direct vision to guide intubation. However, no study has evaluated the performance of SaCoVLM™ video laryngeal mask (VLM) intubation and i-gel combined with flexible bronchoscopy (FB)-guided intubation in airway management during general anesthesia.

**Methods:**

A total of 120 adult patients were randomly allocated into the SaCoVLM™ group (Group S) and i-gel group (Group I). After induction of general anesthesia, guided tracheal intubation under direct vision of the SaCoVLM™ was conducted in Group S, while Group I received FB-guided tracheal intubation using the i-gel. The success rate of SAD placement, first-pass success rate of guided tracheal tube placement, and total success rate in both groups were recorded. The time for SAD placement, time for guided tracheal intubation, total intubation time (time for SAD placement and intubation), glottic exposure grading and postoperative intubation complications (i.e., dysphagia, hoarseness, pharyngalgia, etc.) of both groups were also compared.

**Results:**

The first-time success rate of SAD placement was 98% in two groups. The first-pass success rate of guided endotracheal intubation was 92% in Group S and 93% in Group I (P = 0.74 > 0.05). The total intubation time was 30.8(± 9.7) s and 57.4(± 16.6) s (95% CI = -31.5 to -21.7) in Group S and Group I, respectively (P < 0.01). The total complication rate was 8% in Group S and 22% in Group I (P < 0.05). The laryngeal inlet could be observed in the S group through the visual system of SaCoVLM™. No dysphagia or hoarseness was reported.

**Conclusion:**

SaCoVLM™ can reveal the position of laryngeal inlet, thus providing direct vision for tracheal intubation. SaCoVLM™ -guided intubation is faster, and does not rely on FB, compared to i-gel combined with FB-guided intubation. Besides, SaCoVLM™ has a lower post-intubation complication rate.

**Trial registration:**

Chinese Clinical Trials Registry (ChiCTR2100043443); Date of registration: 18/02/2021.

## Introduction

**Fig. 1:**
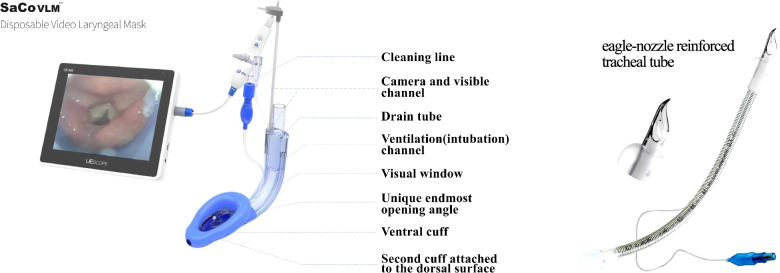
SaCoVLM™ disposable video laryngeal mask and eagle-nozzle reinforced tracheal tube

The 2015 Difficult Airway Society guidelines state that when a difficult airway is unanticipatedly encountered and initial laryngoscopic intubation fails, a supraglottic airway device (SAD) should be placed to ensure ventilation and maintain oxygenation, followed by tracheal intubation via the SAD [1, 2]. SAD has established its role in the difficult airway algorithms and resuscitation guidelines, as a rescue airway device to “buy time” or as a conduit in guiding the TT into the trachea [3, 4]. The SAD can be used for blind intubation or combined with flexible bronchoscopy (FB)-guided intubation in a predicted difficult airway [5–7], However, considering the complications of blind insertion, it is not recommended in this article [8, 9]. SaCoVLM™ video laryngeal mask (VLM, Zhejiang UE Medical Corp,Add: No.8, Youyi Road, Baita Economic Develop Zone, Xianju, Zhejiang, China) is a newly-developed SAD combines the features of double lumen SAD and intubation type SAD (Fig. 1) [4, 10]. SaCoVLM™ can provide a direct vision to guide intubation. FB is not needed in SaCoVLM™-guided intubation, making it more economical and applicable in clinical practice. At present, i-gel (Intersurgical, Berkshire, UK), as a second-generation SAD, is often used for intubation because its ventilation tube has a wide inner diameter which allows the passage of an ETT [11–13]. Previous studies have shown that tracheal intubation using an i-gel in combination with FB provides good visualization of the glottis, with a high success rate of first-pass intubation [14].

This study aimed to explore the success rate of endotracheal intubation using SaCoVLM™ as an intubation conduit. If the success rate of SaCoVLM™-guided intubation is not lower than that of i-gel combined with FB-guided intubation, SaCoVLM™ can be recommended in clinical practice and does not rely on FB for visual intubation.

## Data and methods

### General information

This is a prospective randomized controlled trial approved by the Beijing Hospital Clinical Research Ethics Committee (2020BJYYEC-264–02) and registered in the Chinese Clinical Trials Registry (ChiCTR2100043443; Date of registration: 18/02/ 2021). The trial was registered prior to patient enrollment. The study was conducted between 01/03/2021 and 30/08/2021. This trial adhered to the Consolidated Standards of Reporting Trials (CONSORT) guidelines.

The trial was approved by the appropriate Institutional Review Board (IRB), and written informed consent was obtained from all subjects.

### Randomization method

Randomization was performed using SPSS 23.0 software to obtain grouping information, and random sequences were placed in sealed, opaque envelopes. After determining that the subjects met the enrollment criteria, the envelope was opened to obtain the grouping information and the anesthesiologist was informed of the grouping.

### Inclusion and exclusion criteria

A total of 120 patients undergoing surgery under general anesthesia with endotracheal intubation were screened and randomized into SaCoVLM™ group (Group S) and i-gel group (Group I). Inclusion criteria: understanding the purpose of this study and signing the informed consent; age ≥ 18 years, males or females, BMI 16–35 kg/m^2^, American Society of Anesthesiologists (ASA) class I-II; intending to undergo elective general anesthesia tracheal intubation; operative time less than 4 h. Exclusion criteria: restricted mouth opening (Mouth opening < 2 cm); upper airway tumors, abscesses, foreign bodies or airway stenosis; requiring one-lung ventilation for thoracic surgery.

### Induction of anesthesia

All patients were routinely fasted for 8 h and abstained from drinking for 6 h. After admission, the patient’s peripheral venous access was opened. Electrocardiogram (ECG), heart rate (HR), blood pressure (BP), peripheral oxygen saturation (SpO_2_), end-expiratory carbon dioxide (P_ET_CO_2_), and bispectral index (BIS) were monitored by Grager (Draeger Medical Inc, 3135 Quarry Road Telford, PA, USA). The appropriate size of SAD and eagle-nozzle reinforced tracheal tube (Fig. 1) (Well Lead Medical Co, Ltd, Guangzhou, China) were chosen according to the patient’s weight. Size 3 VLM with 7^#^ tracheal tube was used for patients weighing 30–50 kg, size 4 VLM with 7.5^#^ tracheal tube for patients weighing 50–70 kg, and size 5 VLM with 8^#^ tracheal tube for patients weighing 70–90 kg. The patient was given pre-oxygenation with a face mask (pre-oxygenation: the mask was placed on the face, and 100% pure oxygen (5L/min) was given continuously for 5 min; the pre-oxygenation was completed when the oxygen concentration reached 90% at the end of respiration.), sufentanil (0.2–0.5 μg/kg), propofol (2 mg/kg), and cis-atracurium (0.2 mg/kg) for induction. The patients was ventilated with mask ventilation after BIS was below 60.

### Airway management

The SAD was placed after the mandibular joint was relaxed. SaCoVLM™-guided intubation: slide the SaCoVLM™ from the oral midline along the palatopharyngeal curve in an arc until meeting resistance and reaching the upper esophageal sphincter; inflate the cuff (intracapsular pressure < 60 cmH_2_O), adjust the mask along the palatopharyngeal curve using the up-down maneuver to optimally expose the glottis, fix the mask, inflate the cuff to 60 cmH_2_O (1 cmH_2_O = 0.098 kpa). Cuff inflation pressure was detected by a hand-held manometer (VBM, SULZ, GERMANY). The glottic exposure grade of SaCoVLM™ was recorded at this time. The FB was passed through the SaCoVLM™ ventilator and the glottic exposure grade was recorded at the end of the ventilation tube opening. After the FB was removed, a tracheal tube connected to the breathing circuit (continuous oxygen administration, 5 L/min pure oxygen) was passed through the SaCoVLM™ ventilation tube, and the tracheal tube was placed into the trachea under a direct vision.

i-gel combined with FB-guided intubation: The i-gel was lowered from the midline of the mouth along the arc of the palatopharyngeal curve until encountering resistance and reaching the upper esophageal sphincter. The SAD was connected to the respiratory circuit and ventilation was manually controlled. The successful placement of the SAD was verified by the presence of thoracic movements in a normal tidal volume, no air leakage sound from the SAD, and more than two regular end-expiratory carbon dioxide waveforms. FB with a ETT loaded on its upper shaft was passed through the i-gel ventilation tube, and the glottic exposure grade at the end of the ventilation tube opening was recorded. The FB was placed approximately 3 cm above the carina below the glottis, and the tracheal tube was rotated along the FB and pushed into the trachea. Then, the FB was retracted halfway through the endotracheal tube and then re-entered along the endotracheal tube, simultaneously keeping the carina visible to ensure successful intubation.

The cuff was inflated after completion of endotracheal intubation. Successful intubation can be confirmed by presence of more than two consecutive end-expiratory carbon dioxide waveforms during continuous ventilation through the endotracheal tube. If the SaCoVLM™ failed to guide intubation under the direct vision, the adjustment plan was carried out: (1) move the mask body slightly to adjust the glottic exposure; (2) inflate the cuff to elevate the mask body; (3) assist with extracorporeal laryngeal manipulation (including laryngeal compression, left laryngeal push, right laryngeal push); (4) assist with an elastic bougie; (5) assist with a FB. If successful intubation was not achieved within 120 s or the peripheral oxygen saturation was < 92%, the intubation was declared failed. The SAD was retained after intubation and removed together with the tracheal tube at the end of the operation. In all cases, the SAD was placed for no more than three times during the operation, and if good ventilation was not obtained by adjusting the mask, the SAD placement was considered a failure. Tracheal intubation via the SAD was performed for no more than three times; otherwise, it was considered a failure, and other clinically available methods, such as direct laryngoscopy or video laryngoscopy, were used [1]. All the procedures were completed by an anesthesiologist experienced in airway management.

**Fig. 2:**
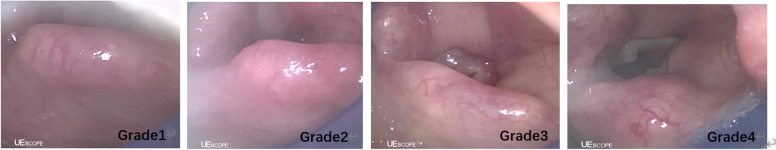
VLM Glottic exposure grades. Grade 1: visualization of the lateral part of the right aryepiglottic fold and part of the laryngeal inlet, and SpO2 > 98% after VLM ventilation; Grade 2: visualization of the bilateral aryepiglottic fold and part of laryngeal inlet, and SpO2 > 98% after VLM ventilation; Grade 3: visualization of all laryngeal inlet and posterior glottis; Grade 4: visualization of the whole glottis

We divided the glottic exposure into four grades under SaCoVLM™ (Fig. 2). Grade 1: visualization of the lateral part of the right aryepiglottic fold and part of the laryngeal inlet, and SpO2 > 98% after VLM ventilation; Grade 2: visualization of the bilateral aryepiglottic fold and part of laryngeal inlet, and SpO2 > 98% after VLM ventilation; Grade 3: visualization of all laryngeal inlet and partial glottis; Grade 4: visualization of the whole glottis. Brimacombe and Berry fibreoptic score [15] were as follows: Grade 1: no visualization of the glottic entrance; Grade 2: visualization of glottis and the lingual surface of epiglottis; Grade 3: visualization glottis and the laryngeal surface of the epiglottis. Grade 4: full visualization of the glottis only [10].

### Anesthesia maintenance

The anesthesia machine was employed for intermittent positive pressure ventilation: fresh air flow 2–5 L/min, VT 6 ml/kg, RR 12–15 times/min, PEEP 5 cmH_2_O, inspiration-expiration ratio 1:2, control EtCO_2_ at 35–45 mmHg. Anesthesia was maintained using target-controlled propofol 2.5–3.5 μg/ml with remifentanil 3–4 ng/ml, BIS control between 40–60, and intermittent additional cis-atracurium.

### Observation index

The primary outcome was the success rate of tracheal intubation using the SaCoVLM™ as an intubation conduit. The secondary outcomes included total intubation time (time for SAD placement and intubation), glottic exposure grading under the FB, glottic exposure grading under SaCoVLM™, time for SAD placement, number of SAD placement attempts, number of intubation attempts, peripheral oxygen saturation before and after intubation and incidence of pharyngalgia, bleeding, hoarseness, and dysphagia.

Time for SAD placement was recorded from the moment when the tip of the SAD touched the incisors to the moment when the second end-expiratory carbon dioxide waveform expiratory upstroke was observed with SAD ventilation after successful SAD placement. Time for intubation using the SaCoVLM™ as the intubation conduit was recorded from the moment when the tip of the endotracheal tube touched the VLM vent opening to the moment when the second end-expiratory carbon dioxide waveform expiratroy upstroke was observed with ETT ventilation. Time for intubation using the i-gel as the intubation conduit was recorded from the moment when the lens of FB with a ETT loaded touched the SAD vent opening to the moment when the second end-expiratory carbon dioxide waveform expiratroy upstroke was observed with ETT ventilation. The time for intubation was recalculated if the first intubation failed and the intubation had to be repeated. This time was observed and recorded by the anesthesia assistant in the study.

### Statistical analysis

The sample size was calculated based on a non-inferiority study with the first-pass success rate of 91% for i-gel combined with FB-guided tracheal intubation in the study by Pavel Michalek et al. [14]. We set a sample size of 120 cases. All data were analyzed using SPSS 26.0 statistical software, and measurement data were expressed as mean ± standard deviation ($\overline{\mathrm{x} }$ ± s). The 95% confidence intervals (95%CI) for difference in the intubation time and the total intubation time between two groups were also calculated. X^2^ test was used in the graded data. The general information of patients and intubation time between groups were analyzed by independent sample t-test. *P* < 0.05 was considered a significant difference.

## Results

There was no statistical difference in age, gender, BMI, mouth opening, thyromental distance, neck circumference, Mallampati classification, upper lip bite classification, and ASA classification between the two groups (Table 1).

**Table 1:** Physical characteristics and airway assessment

	**SaCoVLM™ (*n* = 60)**	**i-gel (*n* = 60)**		***P* value**
Age (years)	55.1 ± 14.1	57.0 ± 11.5		*P* = 0.42
Sex (female)	31(52%)	31(52%)		*P* = 1.00
BMI (kg.m^−2^)	23.8 ± 3.2	24.4 ± 3.2		*P* = 0.37
Mouth opening (mm)	55.8 ± 9.9	55.9 ± 9.2		*P* = 0.92
Thyromental distance (mm)	75.3 ± 11.9	75.81 ± 2.1		*P* = 0.79
Cervical circumference(mm)	348.3 ± 29.1	348.5 ± 47.8		*P* = 0.97
Mallampati score				
1	35(58%)	31(52%)		*P* = 0.63
2	21(35%)	26(43%)		
3	4(7%)	3(5%)		
Upper lip bite test				
1	42(70%)	45(75%)		*P* = 0.82
2	17(28%)	14(23%)		
3	1(2%)		1(2%)	
ASA physical status				
1	39(65%)		34(57%)	*P* = 0.35
2	21(35%)		26(43%)	

Patients were intubated via either SaCoVLM™ or i-gel

*ASA* American Society of Anesthesiologists

The SAD was successfully placed in 120 patients. One case in each group required a second SAD placement. The first-time success rate of SAD placement was 98% in both groups, and the first-pass success rate of guided tracheal intubation was 92% in Group S and 93% in Group I (*p* > 0.05). There was no statistically significant difference in the success rate of the first SAD placement and the first intubation between Group S and Group I (Table 2). In Group S, 55 cases were successfully intubated in the first time, 4 cases in the second time, and 1 case in the third time. In Group I, the first tracheal intubation was successful in 56 cases, the second in 3 cases, and the failure in 1 case which was then converted to laryngoscope-assisted tracheal intubation.

**Table 2:** Results of intubation via SaCoVLM™ or i-gel

	**SaCoVLM™ (*n* = 60)**	**i-gel (*n* = 59)**	***P* value**	**Mean(95%Cl)** **difference**
Successful SAD placement				
First attempt	59(98%)	58(98%)		
Second attempt	1(2%)	1(2%)	*P* = 0.99	
SAD placement time (s)	14.6 ± 3.5	10.9 ± 3.2	*p* < 0.001	3.7(2.5 to 4.9)
Successful ETT insertion				
First attempt	55(92%)	56(93%)	*P* = 0.74	
Second attempt	4(6%)	3(5%)		
Third attempt	1(2%)	0(0)		
Fail	0(0)	1(2%)		
Intubation time (s)	16.3 ± 8.7	47.2 ± 15.7	*p* < 0.001	-30.9(-35.5 to -26.3)
total intubation time (s)	30.8 ± 9.7	57.4 ± 16.6	*p* < 0.001	-26.6(-31.5 to -21.7)
Pre-intubation SpO_2_(%)	97.8 ± 1.3	98.1 ± 1.2	*p* = 0.22	
Post-intubation SpO_2_(%)	97.8 ± 1.2^a^	97.7 ± 1.1^a^	*p* = 0.70	

Total ETT insertion time include SAD placement time and ETT insertion time

*SAD* supraglottic airway device, *s* second, *ETT* endotracheal tube time, *SpO2* peripheral oxygen saturation

^a^There was no significant difference in SpO2 between the two groups before and after intubation

The total intubation time and intubation time in Group S were shorter than those in Group I (30.8[± 9.7] s vs 57.4[± 16.6] s [95% CI = -31.5 to -21.7], 16.3[± 8.7] s vs 47.2[± 15.7] s [95% CI = -35.5 to -26.3], *p* < 0.001). The SAD placement time in Group S was longer than that in Group I (14.6[± 3.5] s vs. 10.9[± 3.2] s [95% CI = 2.5 to 4.9], *p* < 0.001). There was no statistically significant difference between the two groups in terms of the glottic exposure grading under the FB (*p* > 0.05) (Table 3). In group S, the glottis exposure grade was recorded by FB and SaCoVLM™. The results showed that the laryngeal inlet was visualized by SaCoVLM™ in all the cases, and the glottis could be visualized partially or completely using FB at the end of the ventilation tube opening (Table 3). The total complication rate related to the SAD and the use of a tracheal tube via the SAD was 8% in Group S and 22% in Group I (*p* < 0.05) (Table 4). The incidence of postoperative intubation complications was lower in Group S, and the between-group difference was statistically significant. There was no significant difference in peripheral oxygen saturation between the two groups before and after intubation (Table 2).

**Table 3:** FB classification via the end of the ventilation tube opening of SaCoVLM™ or i-gel and glottic exposure grading under the SaCoVLM™ and flexible bronchoscopy in group S

	**SaCoVLM™** **FB classification** **group(*n* = 60)**		**i-gel** **FB classification** **group(*n* = 60)**	***P* value**	**SaCoVLM™** **SaCoVLM™ classification** **group(*n* = 60)**
Grade 1		0 (0)	3 (5%)	*P* = 0.18	4 (7%)
Grade 2		4 (7%)	7 (12%)		8 (13%)
Grade 3		14 (23%)	9 (15%)		21 (35%)
Grade 4		42 (70%)	41 (68%)		27 (45%

Brimacombe and Berry flexible bronchoscopy (FB classification) is as follows: Grade 1: no visualization of the glottic entrance; Grade 2: visualization of glottis and the lingual surface of epiglottis; Grade 3: visualization glottis and the laryngeal surface of the epiglottis. Grade 4: full visualization of the glottis only

SaCoVLM™ grading (SaCoVLM™ classification):Grade 1: visualization of the lateral part of the right aryepiglottic fold and part of the laryngeal inlet, and SpO2 > 98% after VLM ventilation; Grade 2: visualization of the bilateral aryepiglottic fold and part of the laryngeal inlet and SpO2 > 98% after VLM ventilation; Grade 3: visualization of all laryngeal inlet and posterior glottis;Grade 4: visualization of the whole glottis

**Table 4:** Comparison of intubation complications between the SaCoVLM™ and i-gel

	**SaCoVLM™ group (*n* = 60)**	**i-gel group (*n* = 59)**		***P* value**
Blood-stained SAD	3(5%)		5(8%)	*P* = 0.45
Blood-stained tube	1(2%)		7(12%)	*P* = 0.03
Pharyngalgia	3(5%)		6(10%)	*P* = 0.29
Hoarseness	0(0)		0(0)	
Dysphagia	0(0)		0(0)	
Total complication rate	5(8%)		13(22%)	*P* = 0.037

## Discussion

In this study, we compared the effectiveness of two brands of SADs to guide tracheal intubation. The results showed no significant difference in the total success rate of intubation between the two groups, and the tracheal intubation time in Group S was shorter than that in Group I. The laryngeal inlet could be visualized in Group S with its own visual system. No serious intubation complications occurred in the two groups, and the total complication rate related to the SAD and the use of a tracheal tube via the SAD in Group S was lower. The success rates in the first placement of both SaCoVLM™ and i-gel were 98%; the success rates of first guided tracheal intubation were 92% and 93%; the total intubation success rates were 100% and 98%, respectively. Some studies have concluded that the success rate of FB tracheal intubation through i-gel was 91–100% [6, 16]. C. Mendonca et al. compared FB-guided tracheal intubation through i-gel and LMA Protector, and found that the success rate of first intubation was 98% and the total success rate was 100% [6]. These results are consistent with the results of this study. A meta-analysis in 2014 found that the glottic view via an i-gel was significantly larger than the LMA Classic [17]. In this study, there was no statistically significant difference in the grade of glottic exposure under the FB between Group S and Group I. Thomas Metterlein et al. compared four types of SAD: i-gel, Unique, Supreme, and Aura-Once, all using FB to observe the ratios of good glottis exposure. The good glottic exposure ratios of these four SADs were 70%, 90%, 83%, and 90%, respectively. Fully or partially exposed glottis was defined as good glottis exposure in the study of Thomas Metterlein [12]. According to this definition, the good glottic exposure ratios of SaCoVLM™ and i-gel under FB in this study were 93% and 83%, respectively. This study showed that both SaCoVLM™ and i-gel could achieve a good alignment with the laryngeal inlet after placement. In addition, SaCoVLM™ not only aligns well with the glottis, but also visualizes the laryngeal inlet without FB. The tracheal intubation is performed under a direct vision, while maintaining continuous ventilation to avoid intubation complications caused by blind intubation. The results are similar to those of other studies on visual SADs [18–20].

The SAD placement time in Group S was longer than that in Group I. This is because that the SaCoVLM™ uses an inflatable cuff; whereas the i-gel uses a thermoplastic non-inflatable cuff, so it takes it a longer time to inflate the VLM cuff after insertion. The intubation time in Group S was shorter than that in Group I. This might be explained by the fact that through the camera on the right side of the SaCoVLM™ vent, we could directly see the relative position of endotracheal tube and glottis, just like using a laryngoscope to complete intubation [21, 22]. In contrast, the intubation in Group I was more complex, and a longer learning curve was needed in the use of FB. Baker PA et al. found that the failure rate of FB-guided tracheal intubation was higher and it took a longer time master FB operation for anesthesiologists lacking experience or training [23]. S. Sreevathsa et al. compared the intubation time of LMA Fastrach™ and LMA CTrach™, which were 84 ± 32 s and 53 ± 21 s, respectively [7].  The intubation time of LMA CTrach™ was significantly longer than that of SaCoVLM™, which may be due to the unique endmost opening angle of SaCoVLM™, which facilitates the guidance of the endotracheal tube into the glottis and reduces some alignment adjustments (Fig. 1). In addition, FB guided intubation does not offer a full visualization. In the FB intubation, the relative position of the ETT and the glottis cannot be seen, so it is also a kind of blind insertion. Therefore, resistance is often encountered during the insertion of the ETT, which requires blind adjustment; this prolongs the intubation time. So, the intubation time in Group S was significantly shorter than that in Group I.

The incidence of postoperative intubation complications in Group I was significantly higher than that in Group S. In using FB to guide intubation, FB was inserted into the trachea first, and then the endotracheal tube was rotated and pushed into the trachea along the FB. During this process, the relative position of the tube and glottis could not be observed. Therefore, friction between the opening of the tube and the glottis may damage surrounding tissues, triggering strong response of the patient [24]. SaCoVLM™ can show the relative position of endotracheal tube and glottis, as a visual laryngoscope does. Any misalignment could be avoided by pushing the throat or inflating/deflating the cuff of the mask body. Therefore, the end of the tube can be appropriately positioned to reduce the damage to the tissues around the throat. This reason may explain the low incidence of intubation complications in Group S.

In one patient in Group S, the first intubation failed, because the epiglottis was wide and long enough to cover the laryngeal inlet. Therefore, the volume of air in the inflated mask cuff was increased to expand the space in the hypopharyngeal cavity and improve glottis exposure from Grade 1 to Grade 3. The overinflated mask cuff further elevated the epiglottis root and improved glottis exposure. Finally, the third intubation was successful. In another 4 cases, the first intubation failed because the tip of the tube was lower than the interarytenoid notch and could not enter the glottis. To make the second intubation successful, we adjusted the relative position of the tube and glottis by up-down maneuver as well as push at left and right larynx.

For one female patient in Group I (BMI = 32 kg/m^2^), i-gel size 5 was selected based on her body weight, but intubation through SAD failed. After three attempts, the endotracheal tube could not pass by the epiglottis, and the tube was successfully intubated through a laryngoscope with the assistance of extracorporeal laryngeal pushing. This event might be related to the thermoplastic structure of i-gel, which is designed according to the anatomical structure of the pharynx. The volume of the mask body was fixed, so the hypopharyngeal cavity could not be changed. After insertion, the epiglottis root might be compressed, consequently folding back the epiglottis to cover the laryngeal inlet. In the study by Lee et al., the i-gel with a jelly-like thermoplastic structure ended up with a higher incidence of epiglottis reflex and higher airway resistance than the air-Q mask with an inflatable capsule [25]. If the epiglottis reflexes with SaCoVLM™, the hypopharyngeal space can be increased by inflating the capsule, and the glottis exposed by lifting the epiglottis root. At the same time, up-down maneuver can be performed to best expose the glottis under visual view [22].

There are still some limitations in this study. First, all operations were performed by a single operator. This avoids operator-related error in the glottic exposure classification and leads to a higher success rate of SAD placement and intubation. But external validity is needed. Second, the peripheral oxygen saturation in both groups remained above 95% throughout the intubation procedure. It is not clear whether there is a difference in oxygenation between patients with poor oxygen reserve receiving SaCoVLM™ continuous oxygenation-guided intubation and i-gel combined with visualization. Third, the introduction describes intubation through an SAD as a part of difficult airway management. This study included only suspected easy airways. Future studies on suspected difficult airways are needed. Fourth, in this study, the two devices were transitionally used from anesthesia induction to completion of endotracheal intubation, rather than for a long-term maintenance of ventilation, so ventilation parameters of the two devices were not described in detail.

## Conclusion

SaCoVLM™ can be visually intubated without relying on FB. The success rates of SaCoVLM™-guided intubation and i-gel combined with FB-guided intubation show no significant difference. The SaCoVLM™-guided intubation is faster and less complicated than that with i-gel. In addition, the SaCoVLM™ allows continuous oxygen supply during intubation.

## Data Availability

The datasets generated and/or analysed during the current study are not publicly available due to laws about patents but are available from the corresponding author on reasonable request.

## Abbreviations

SaCoVLM^TM^: SaCoVLM^TM^ video laryngeal mask
SAD: Supraglottic airway device
VLM: Video laryngeal mask
FB: Flexible bronchoscopy
BMI: Body mass index
ASA: American Society of Anesthesiologists;
ECG: Electrocardiogram
HR: Heart rate
BP: Blood pressure
SpO_2_: Peripheral oxygen saturation
P_ET_CO_2_: End-expiratory carbon dioxide
BIS: Bispectral index
ETT: Endotracheal tube time;
CONSORT: Consolidated Standards of Reporting Trials
